# Podocytic PKC-Alpha Is Regulated in Murine and Human Diabetes and Mediates Nephrin Endocytosis

**DOI:** 10.1371/journal.pone.0010185

**Published:** 2010-04-16

**Authors:** Irini Tossidou, Beina Teng, Jan Menne, Nelli Shushakova, Joon-Keun Park, Jan U. Becker, Friedrich Modde, Michael Leitges, Hermann Haller, Mario Schiffer

**Affiliations:** 1 Division of Nephrology, Department of Medicine, Hannover Medical School, Hannover, Germany; 2 Department of Pathology, Hannover Medical School, Hannover, Germany; 3 The Biotechnology Centre of Oslo, University of Oslo, Oslo, Norway; University of Birmingham, United Kingdom

## Abstract

**Background:**

Microalbuminuria is an early lesion during the development of diabetic nephropathy. The loss of high molecular weight proteins in the urine is usually associated with decreased expression of slit diaphragm proteins. Nephrin, is the major component of the glomerular slit diaphragm and loss of nephrin has been well described in rodent models of experimental diabetes as well as in human diabetic nephropathy.

**Methodology/Principal Findings:**

In this manuscript we analyzed the role of PKC-alpha (PKCα) on endocytosis of nephrin in podocytes. We found that treatment of diabetic mice with a PKCα-inhibitor (GÖ6976) leads to preserved nephrin expression and reduced proteinuria. *In vitro*, we found that high glucose stimulation would induce PKCα protein expression in murine and human podocytes. We can demonstrate that PKCα mediates nephrin endocytosis in podocytes and that overexpression of PKCα leads to an augmented endocytosis response. After PKC-activation, we demonstrate an inducible association of PKCα, PICK1 and nephrin in podocytes. Moreover, we can demonstrate a strong induction of PKCα in podocytes of patients with diabetic nephropathy.

**Conclusions/Significance:**

We therefore conclude that activation of PKCα is a pathomechanistic key event during the development of diabetic nephropathy. PKCα is involved in reduction of nephrin surface expression and therefore PKCα inhibition might be a novel target molecule for anti-proteinuric therapy.

## Introduction

The activation of protein kinase C (PKC) in the kidney is a well known pathway of the diabetic milieu. However the influence and function of PKC on podocyte actions has so far not been studied systematically. The 10 PKC family members are products of distinct genes and differ in their biochemical properties, tissue specific expression and intracellular localization [1]. PKCs are stimulated in response to cytokines as well as various stressors including osmotic stress and high glucose [2]. PKCs can be subclassified into three groups: The conventional isoforms (PKCα, βI, βII and γ) require a lipid cofactor (phosphatidylserine (PS), Ca^2+^ or 1,2-diacylglycerol (DAG)) for activation; the novel isoforms (PKCδ, ε, η, θ, µ) are Ca^2+^ independent and the atypical isoforms (PKCι/λ and ζ) require only PS [3]. With the exception of the atypical PKC-isoform, PKCι/λ [4], the isoform-specific knockout does not lead to a distinct renal phenotype, however in conditions with aberrant PKC-activation, such as diabetes or unilateral ureteric obstruction, the role of different PKC-isoforms becomes apparent. We previously described that PKCα deficient mice show a better outcome after streptozotocin (STZ) induced diabetes with less proteinuria and preserved nephrin expression [5], [6]. To study the involvement of PKCα in proteinuria development in diabetic nephropathy, we treated mice after streptozotocin induced diabetes with a synthetic PKCα inhibitor (GÖ6976), which could prevent proteinuria development and led to preserved nephrin expression. Biochemical analysis in wildtype and PKCα deficient podocyte lines revealed a central role for PKCα in endocytosis of the slit diaphragm component nephrin. This endocytosis mediation could account for the preserved nephrin expression *in vivo*. Since we observed increased expression of PKCα in podocytes in renal biopsies of patients with diabetic nephropathy we conclude that PKCα inhibition might be a useful therapeutic option to prevent proteinuria development.

## Materials and Methods

### Ethics Statement

Animal work was conducted according to the guidelines of the American Physiologic Society and was approved by Institutional Animal Care and Use Committee of Hannover Medical School and the animal welfare authorities of lower saxony. All efforts were made to minimize the number of animals used and their suffering. The mice received a standard diet with free access to tap water.

### Clinical renal biopsy samples

Renal tissue was obtained from the archives of the Department of Pathology, Hannover Medical School. Paraffin-embedded specimens of normal kidney from nephrectomies performed for tumor, and renal biopsy samples from adults with diabetic nephropathy were included in this study. Renal biopsies were used in accordance with the ethical standards of the Hannover Medical School and with the declaration of Helsinki of 1975, as revised in 1983. All patients consented at hospital admission to experiments on their anonymized archived tissue samples.

### Type I diabetes model

Type I diabetes was induced in 8-week-old SV129 male mice obtained from Charles River Laboratories (Sulzfeld, Germany) by intraperitoneal injection of streptozotozin (STZ) (Sigma-Aldrich, St Louis, MO) at 125 mg/kg/day in 50 mmol/l sodium citrate puffer (pH 4.5) on days 1 and 4. The blood glucose level was measured with the Glucometer Elite (Bayer, Leverkusen, Germany) every other day. Approximately 80 % of the animals were diabetic 14 days after the first injection. Only animals which have stable blood glucose level more than 15 mmol/l and no loss of weight more than 20% within the first days after onset of diabetes were used for following experiments. The plasma glucose level was measured further once weekly. The diabetic mice were distributed into two groups (n = 23 per group). One group of diabetic mice was treated daily with GÖ6976 (LC Laboratories, Woburn, Massachusetts) in dose 100 µg/8 µl DMSO subcutaneously, the second group was injected daily with 8 µl DMSO alone. Group of nondiabetic mice (n = 16) at 8 weeks of age were assessed for blood glucose and albuminuria and served as controls.

### Albuminuria

Animals were placed in metabolic cages for 24-hour urine collection. Albumin concentration in appropriately diluted urine samples was measured using the Albuwell M Assay Kit (Exocel, Philadelphia, PA, USA) according to the manufacturer's instructions.

### Statistics

Data are shown as mean ± SD and were compared by *Student's t-test*. Data analysis was performed using Excel statistical software. The data for albumin excretion were compared by ANOVA, and the Bonferroni multiple comparison test was used as posttest. Data analysis was performed using InStat. Significant differences were accepted when *P*<0.05.

### Antibodies and Inhibitors

Antibodies that were used for Western blotting, immunohistochemical and immunofluorescence studies were: rabbit anti-PKCα, rabbit anti-β-tubulin, rabbit anti-PICK1 (Santa Cruz Biotechnology, Santa Cruz, CA), guinea pig polyclonal anti-nephrin (for immunohistochemical studies) (PROGEN, Heidelberg, Germany), ectodomain rabbit anti-nephrin (for endocytosis assays) (Santa Cruz Biotechnology, Santa Cruz, CA) goat anti-podocalyxin (R&D, Minneapolis, USA, ), rabbit anti-flag, rabbit anti-myc, rabbit anti-GFP (Cell Signaling Technology, Beverly, MA USA), mouse anti-V5 antibody (Serotec, Kidlington, UK), Alexa Fluor 568 goat anti-guinea pig IgG (Invitrogen, Carlsbad, CA, USA). The used PKC inhibitor was the PKCα-inhibitor (GÖ6976) (Calbiochem, Merck, Darmstadt, Germany).

### Isolation of glomeruli, establishment of conditionally immortalized murine podocyte cell lines and podocyte culture

Targeted disruption and generation of PKCα-deficient homozygous mice on a SV129 background is described elsewhere [7]. PKCα-deficient mice were crossed to immorto mice (in BL6/C57 background) and used for generation of immortalized cell lines. Isolation of glomeruli and establishment of PKCα deficient murine podocyte lines was described earlier [8]. Every experimental setup and result was confirmed in 3 different clones of PKCα+/+ and PKCα−/− podocytes. Continuous cultivation of conditionally immortalized mouse podocytes was performed as described by Mundel et al. 1996 [9].

### Western Blot Analysis

To analyze whole cell protein lysates from cultured podocytes, either untreated or treated cells were lysed on ice in RIPA buffer (50 mM Tris pH 7.5, 150 mM NaCl, 0.5 % sodium deoxycholate, 1 % Nonidet P-40, 0.1 % SDS) containing protease inhibitor (Complete mini, Roche, Mannheim, Germany), 1 mM sodium orthovanadate, 50 mM NaF and 200 µg/L okadaic acid. Lysates were centrifuged at 12,000 rpm and aliquots of the supernatants were seperated by 10 % SDS-PAGE and transferred to PVDF membrane (Immobilon-P, Millipore, Bedford, MA, USA). After probing with primary antibodies antigen-antibody complexes were detected with horseradish peroxidase-labeled anti-rabbit and anti-mouse antibodies, respectively, and visualized using enhanced chemiluminescence reagents (Pierce, Rockford, IL, USA) according to the manufacturer's protocol.

### Quantitative PCR (Q-PCR) analysis of cultured cells

Total RNA was prepared from cultured human or murine podocytes using the RNeasy®MiniKit (Qiagen, Hilden, Germany) following the protocol recommended by the manufacturer with an additional step of DNAse digestion (RNase-Free DNase Set; Qiagen). One microgram of total RNA was reverse transcribed using Oligo (dT) 15 and random primers and M-MLV Reverse Transcriptase (Promega, Mannheim, Germany). Quantitative PCR (qPCR) was performed on a Light Cycler 480 (Roche, Mannheim, Germany). The cDNA was amplified using Fast Start Taq Polymerase (Roche Diagnostics, Mannheim Germany), SYBR Green (Molecular probes, Eugene, OR), gene specific primers and the following PCR conditions: 5 min at 95°C, 45 cycles for 10 s at 95°C, 10 s at 60°C and 10 s at 72°C. Specifity of the amplification product was verified by melting curve analysis. The samples were measured as multiplexed reactions and normalized to the constitutive gene human Hypoxanthin-Phosphoribosyl Transferase 1 (HPRT-1). All primers for the listed transcripts were designed using Primer3 software: mPKCα left: AGA ACA GGG AGA TCC AAC CA, mPKCα right: CAA AGT TTT CTG CTC CTT TGC, hPKCα left: ACC ATT CAA GCC CAA AGT GT, hPKCα right: GGC TGT CCT CGT GTG TGA AGA, mNephrin left: GGG GAC CCC TCT ATG ATG A, mNephrin right: TGG GTC CTC ATA TTT GAC CTC, hNephrin left AGG AGC CAT AGG GAA TGG AC, hNephrin right: GCA GAC GGA GCC TTC TTG T.

### Laser microdissection of paraffin embedded tissue, mRNA extraction and Q-PCR

Formalin fixed and paraffin embedded renal biopsies were cut into 4-µm sections. 60 glomeruli of each specimen (n = 5 controls and n = 5 with DN) were micro dissected on a mmi CellCut Plus System (Olympus, Hamburg, Germany) without prior deparaffinization and collected for RNA isolation. The tissue was digested in 50 µL of 20 mg/mL Proteinase K solution (Merck, Darmstadt, Germany), 50 µL of RNA digestion solution (4.2 M guanidinium thiocyanate, 30 mM Tris-HCl of pH 7.6, and 2% sodium N-lauroylsarcosine), and 0.5 µL of 2-mercaptoethanol at 55°C over night immediately. Then 10 µL of 3 M sodium acetate (pH 5.2), 63 µL of Roti-Aqua-Phenol (Roth, Karlsruhe, Germany), and 27 µL of chloroform were added to precipitate RNA. After phase separation through centrifugation, the aqueous phase (95 µL) was carefully removed and added to 96 µL of 2-propanol and 1 µL of glycogen (20 mg/mL; Roche, Mannheim, Germany) as precipitation carrier. The precipitated RNA was then stored at −20°C. cDNA synthesis was performed for 2 hours at 37°C as prescribed in the kit manual of the High Capacity cDNA Reverse Transcription Kit (Applied Biosystems, Massachusetts, USA). cDNA was then pre-amplified using TaqMan® PreAmp Master Mix (2×) (Applied Biosystems, Massachusetts, USA) according to the manual of the manufacturer. Primer pool used for pre-amplification consisted of 6 TaqMan® Assays (Applied Biosystems, Massachusetts, USA), including PKC-alpha, WT1 and two housekeeping genes (β-Gus, and Polymerase R2A). 25 µL of TaqMan® PreAmp Master Mix as well as 12,5 µL of the assay pool were added to 12,5 µL of cDNA and subjected to the following thermocycler program: 95°C for 10 minutes, 14 cycles of 95°C for 15 seconds and 60°C for 4 minutes. Pre-amplified cDNA was stored at −20°C. 5 µL of the pre-amplified cDNA (1∶10 dilution) were added to 15 µL of master mix consisting of 10 µL of Gene Expression Master Mix (Applied Biosystems, Massachusetts, USA), 4 µL of purified water and 1 µL of the respective TaqMan® Gene Expression Assay (Applied Biosystems, Massachusetts, USA). RT-PCR primers were used to amplify PKC-alpha (Hs00176973_m1), WT1 (Hs 01103751_m1), POLR2A (Hs 00172187_m1) and GUS B (Hs 99999908_m1). RT-PCR was performed with a 7500 Fast Real-Time PCR System (Applied Biosystems, Massachusetts, USA). Results are given as relative expression levels.

### Transfection

The day before transfection HEK 293T cells were seeded on 24-well plates for endocytosis assays or on 10 cm-dishes for immunoprecipitation assay. Cells were transfected using Fugene HD transfection reagent (Roche Diagnostics, Mannheim, Germany) according to the manufacturer's protocol. After transfection cells were cultured in normal growth medium for 48 h.

### Immunoprecipitation

HEK 293T cells were transfected like mentioned above. Then the cells were washed carefully with ice-cold PBS on ice. For lysis 900 µl RIPA buffer were added to the cells. The lysate was incubated for 15 min on ice and centrifuged at 14.000 rpm for 15 min at 4°C. 50 µl Flag-beads (50% slurry in Triton buffer) (Sigma, St. Louis, USA) were added to the supernatant and rotated overhead at 4°C for 1 h (up to overnight). After that the beads were centrifuged at 3000 rpm for 1 min at 4°C and washed with RIPA-buffer 5 times. Proteins were eluted by boiling the beads in Laemmli-buffer and separated by SDS polyacrylamide gel electrophoresis. Western blot was performed using the methods mentioned above. For IP-Assay of podocytes 500 µg total cell lysate was incubated with 2 µg anti-nephrin or anti-PICK1 antibody /ml IP-Volume and sepharose-A beads in IP-buffer (25 mM Tris-HCL, pH 7.5, 1 mM DTT, 30 mM MgCL_2_, 40 mM NaCL, 0,5% NP-40 and protease inhibitors) overnight at 4°C. The pellets were washed in IP-buffer (three washing steps in total). The protein was eluated by boiling the beads in Laemmli buffer. Lysates and elutes are analyzed by SDS polyacrylamide gel electrophoresis.

### Immunocytofluorescence-based Endocytosis Assay

For internalization studies, cells were plated in 24-wells on glass slides and differentiated for 10 days. Cells were cooled down at 4°C and incubated with the raft-mediated endocytosis marker Cholera toxin subunit B (Alexa Fluor 488 conjugated) (Invitrogen, Eugene, Oregon, USA) and simultaneously with an ectodomain anti-nephrin antibody and Cy3-conjugated secondary antibody for 1 h in RPMI without FCS at 4°C. After three washes with PBS cells were incubated for 20 min at 4°C or 37°C to induce internalization before fixing them with 4% paraformaldehyde. The pictures were taken with an Inverted-2 Confocal Microscope and Leica Application Suite Software (Leica, Bonn, Germany). Post processing was done with Photo Shop 6.0.

### ELISA-based Endocytosis Assay

HEK 293T cells were transfected with constructs (mNephrin-flag, hNephrin-SV5, pGFP-N1, PKCα-GFP) using the FUGENE-Reagent (Roche, Mannheim, Germany). 24-well plate was coated with poly-L-lysine 1∶1 diluted with H_2_O (Sigma P4707 0,01% Solution, Steinheim, Germany) and incubated overnight at 37°C. The next day the plate was washed once with PBS and transfected HEK-293T cells (6×10^5^ /well) were seeded. About 30 hours after the transfection cells were cooled on ice for 10–15 minutes. The medium was replaced with DFH-medium (1% FCS and 20 mM HEPES in RPMI1640) containing 1∶750 mouse anti-V5 antibody or 1∶200 rabbit anti-flag antibody. The cells were incubated for 30–60 minutes at 4°C and then washed three times with cold DFHI-medium (1% FCS and 25 mM HEPES in RPMI1640). To induce internalization cells were incubated with warm DFHI-medium (1%FCS and 5 mM HEPES in RPMI1640) for 20 minutes at 37°C. The cells were fixed with 3.7% PFA for 15 minutes, washed twice with PBS and kept over night at 4°C. For blocking 2% normal goat serum (Jackson ImmunoResearch, USA) with 0.5% Tween-20 in PBS was used for at least 30 minutes. After washing once with PBS the cells were incubated in RPMI1640-medium with alkaline phosphatase coupled anti-mouse or anti-rabbit antibody (dilution 1∶7.500) for 1 hour and washed three times with PBS, each wash 5–10 minutes. The cells were then incubated with p-Nitrophenyl Phosphate (pNPP: Sigma N2765, Steinheim, Germany, one tablet was resuspended in 20 ml 0,1 M glycine, 1 mM MgCl_2_, 1 mM ZnCl_2_, pH 10,4) for about 1 hour at 37°C or until the solution turned yellow. One aliquot, about 100 µl, from the reaction was transferred in a 96-well-plate and the extinction was measured at 405 nm in a microplate reader. Murine PKCα+/+ and PKCα−/− and human podocytes were differentiated for 10 days. Endocytosis of endogenous nephrin was induced by temperature shift (20 min) or by stimulation with high Glucose medium (30 mM). For high glucose treatment cells were cultured for 1 hour with medium containing 30 mM D-glucose or with medium containing normal glucose concentration supplemented with Mannitol as osmotic control. The cells were cooled down at 4°C to stop endocytosis and nephrin was labeled with an ectodomain anti-nephrin antibody. Endocytosis of nephrin was measured by the same method as mentioned above.

### Immunohistochemistry

After dissection, kidneys were washed with phosphate-buffered saline and immediately frozen in tissue molds containing OCT compound (Tissue Tek). For fluorescent images were collected onto slides, blocked with 10% donkey-serum, stained with the appropriate primary antibody followed by a Cy3-(Jackson Immunoresearch Lab, West Grove USA) or Alexa 488- (Invitrogen GmbH, Karlsruhe, Germany) conjugated secondary antibody. Sections were analyzed using a Zeiss Axioplan-2 imaging microscope with the scientific image processing software AxioVision 4.6 (Zeiss, Jena, Germany). The human slides were stained following a standard peroxidase staining protocol and hematoxylin counterstain. Human renal tissue was obtained from the archives of the Department of Pathology, Hannover Medical School. Paraffin-embedded specimens of normal kidney from nephrectomies performed for tumor (n = 7), and renal biopsy samples from adults with DN (n = 10) were included in this study. All glomeruli were scored for expression of PKCα (n = 74 glomeruli for DN and n = 150 normal glomeruli). All patients with renal disease had significant proteinuria at the time of biopsy. Renal biopsies were used in accordance with the ethical standards of the Hannover Medical School and with the declaration of Helsinki of 1975, as revised in 1983.

## Results

### PKCα is upregulated in podocytes in experimental as well as in human diabetic nephropathy

**Figure 1:**
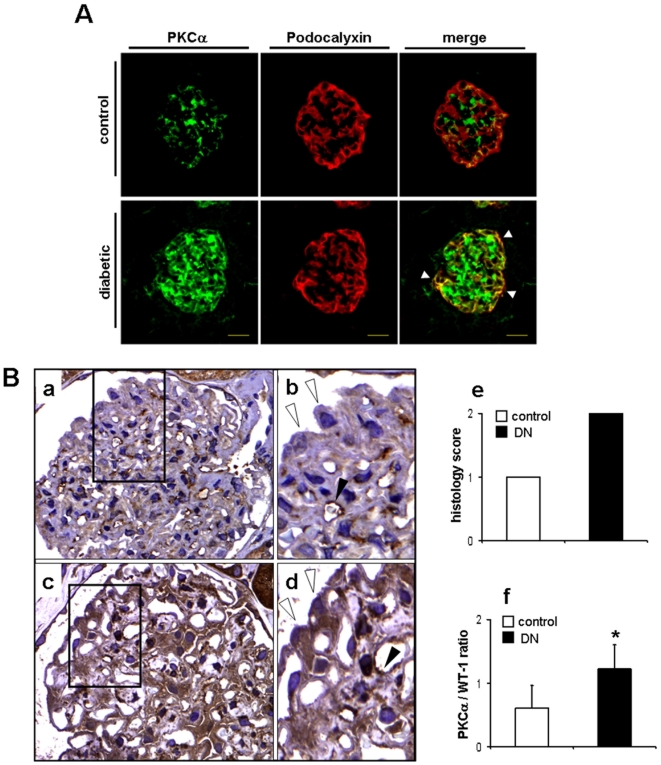
Glomerular PKCα is upregulated in mice and patients with diabetic nephropathy. (A) Immunofluorescence using an anti-PKCα (green) and anti-podocalyxin (red) antibody on frozen kidney cortex sections of control and diabetic mice. White arrowheads in the merged panel depict colocalization of PKCα with podocalyxin. Picture is representative for the majority of glomeruli in control and diabetic mice (n = 5) in each group). (B) Immunohistochemistry using an anti-PKCα antibody shows expression of PKCα in human biopsy samples of healthy control individual (a, b) and a patient with diabetic nephropathy (c, d). Black arrowheads depict expression of PKCα in the glomerular endothelium and open arrowheads depict podocytes in normal and diabetic individual (panel b, d). Bar graphs (e) summarize semiquantitative score of all glomeruli (n = 74 for patients with DN and n = 150 for control cases), indicating 1 for absent or moderate or 2 for strong expression of PKCα in podocytes and mRNA expression of PKCα isolated from control (n = 300) and diabetic (n = 298) human glomeruli in relation to Wt-1 mRNA expression, *p<0.05 compared by *Student's t-test* (f).

**Figure 2:**
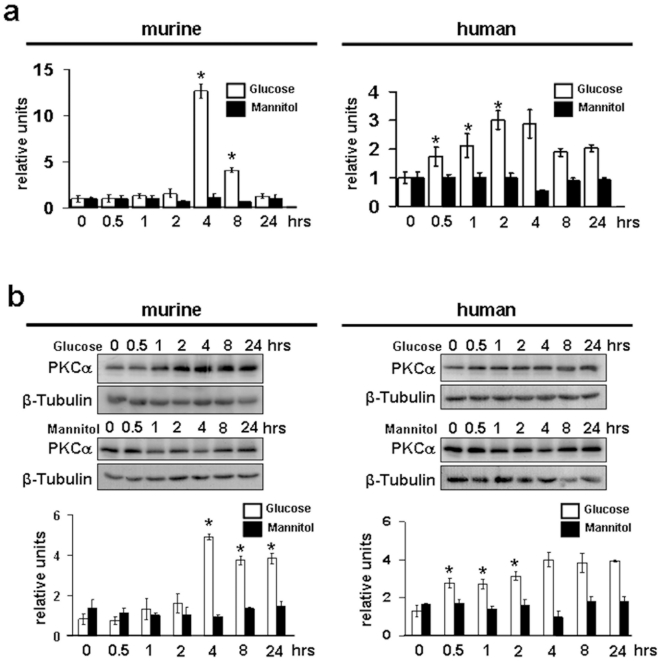
PKCα expression is upregulated by high glucose in murine and human podocytes. Quantitative PCR (a) and Western blot analysis (b) for PKCα in murine and human podocytes depicts induction of protein in a time course experiment after stimulation with high glucose (30 mM) and mannitol as osmotic control for up to 24 hrs (results are representative for 3 independent experiments). Scale bars of quantitative PCR summarizes means of high glucose (white bars) and Mannitol (black bars) of n = 3 independent experiments. (*p<0.01, 0 versus 4, 8 hrs (murine) and *p<0.05 0 versus 0.5, 1, 2 hrs (human) compared by *Student's t-test*. Densitometric analysis summarizes means of PKCα and β-tubulin ratios after high glucose (white bars) and mannitol (black bars) stimulation in *n* = 3 independent experiments. OD was quantified using Quantity One software. *p<0.03, 0 hrs versus 4, 8, 24 hrs (murine), *p<0.04 0 versus 0.5, 1, 2 hrs (human) compared by Student's *t-test*.

First, we wanted to examine whether diabetes leads to changes in PKCα expression in mice and in patients with diabetic nephropathy. When we stained frozen kidney cortex sections of control mice and mice 4 weeks after STZ-induced diabetes, we found a strong upregulation of glomerular PKCα expression (Fig. 1A). Next to a strong upregulation in the endocapillary/mesangial compartment, double stainings with the podocyte marker podocalyxin revealed a strong upregulation in podocytes in the diabetic mice (Fig. 1A). Similarly, when we stained different biopsy samples from patients with features of diabetic nephropathy (n = 10) compared to healthy controls (n = 7), we found that PKCα staining in healthy controls is predominantly visible in the glomerular endothelium (Fig. 1B, panel a, b), whereas podocyte staining was absent. Strikingly, in patients with diabetic nephropathy we can demonstrate an expression of PKCα in glomerular endothelium as well as strong expression in all podocytes (Fig. 1B, panels c,d). This clear difference was detectable in all 10 examined patients. All podocytes displayed a strong PKCα staining, whereas in all examined control glomeruli endothelial staining was evident, but podocytes were rarely detected positive for PKCα. When all glomeruli were semiquantitavely scored with a score of 1 indicating absent or moderate expression and a score of 2 indicating strong expression we could document uniformly an average score of 1 for all examined control glomeruli and an average score of 2 for all examined diabetic glomeruli (Fig.1B, panel e). In addition, when we performed laser capture microscopy and extracted mRNA from control and diabetic human glomeruli we found a significantly increased mRNA expression of PKCα mRNA in the diabetic glomeruli (Fig. 1B, panel f, *p<0.05). To delineate that high concentration of D-glucose (30 mM) would lead to podocytic upregulation of PKCα, we cultured murine and human podocytes for up to 24 hours in the presence of 30 mM D-glucose. We found a significantly upregulated expression of podocytic PKCα on mRNA and protein level. In contrast, mannitol had no significant effect on PKCα expression (Fig. 2, panel a, b). These data indicate, that PKCα expression is upregulated in podocytes in experimental diabetes in mice as well as in human diabetic nephropathy. Moreover, we found *in vitro* that high glucose is a strong regulator of PKCα expression in podocytes. To exclude direct expression effects on nephrin mRNA in murine and human podocytes we performed Q-PCR-analysis of nephrin mRNA in both cells types during the 24 hour time course. We could not detect a transcriptional regulation of nephrin mRNA in both cell types in the presence of 30 mM glucose or mannitol (Supplementary Fig. S1).

### Chemical PKCα inhibition stabilizes nephrin expression and prevents proteinuria in streptozotocin induced diabetes in mice

**Figure 3:**
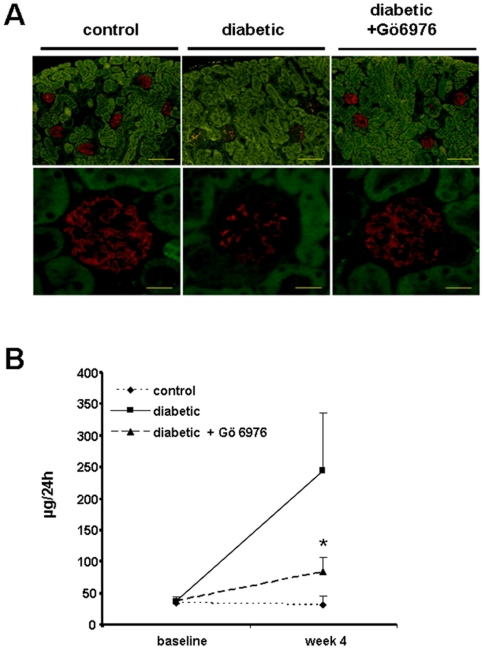
PKCα inhibition or deficiency preserves nephrin expression and reduces proteinuria in vivo. (A) Diabetes was induced in SV129 mice with STZ. Immunofluorescent images show expression of nephrin (red) in control, untreated diabetic or GÖ6976 treated diabetic mice (pictures are representative for the majority of glomeruli in the three different groups) (scale bar upper panels 100 µm, lower panels 20 µm). (B) Graphical summary of urinary albumin excretion in 24 hrs of control (n = 16), diabetic (n = 23) and diabetic mice treated with GÖ6976 (n = 23), *p<0.03 diabetic mice versus GÖ6976 treated diabetic mice.

Since we had previously described preserved nephrin expression and reduced proteinuria in diabetic PKCα deficient mice we wanted to test whether chemical inhibition of PKCα would have a similar protective effect. Our first observation was that under diabetic conditions the glomerular expression of nephrin is downregulated. Strikingly, the treatment of diabetic mice with the PKCα inhibitor GÖ6976 led to preserved nephrin expression (Fig. 3A). When we compared total protein excretion in diabetic mice untreated or treated with GÖ6976 for four weeks, we found a significant reduction of urinary protein levels in the GÖ6976 treated group (67±97.1 µg/24 h, n = 23) compared to untreated diabetic mice (234±240.3/24 h, n = 23, *p<0.03) (Fig. 3B). This treatment success led us to the hypothesis, that inhibition of PKCα would have a direct effect on nephrin expression and slit diaphragm integrity *in vivo* and thus it could be of potential use in proteinuric situations.

### PKCα regulates nephrin endocytosis

**Figure 4:**
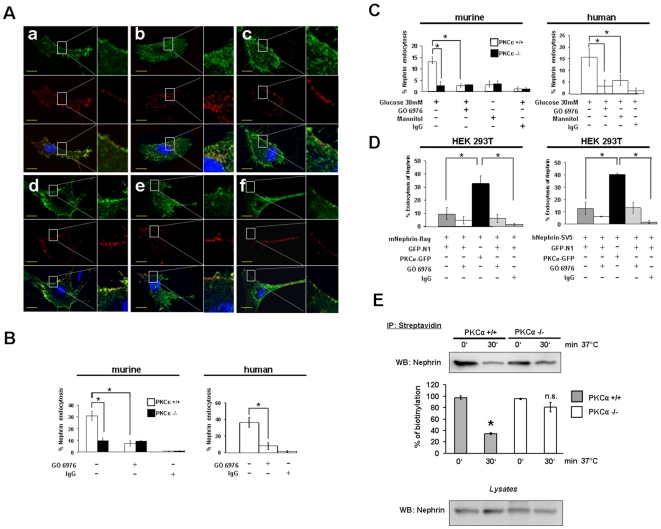
PKCα promotes nephrin endocytosis. (A) PKCα+/+ (a, b, c) and PKCα−/− (d, e, f) podocytes were double labelled with Cholera toxin-B (green) and an ectodomain anti-nephrin antibody detected with an Cy3-labeled secondary antibody (red). The cells are labelled at 4°C and incubated at 4°C (a, d) or shifted to 37°C (b, c, e, f) for 20 min to induce internalization in the absence (b, e) or presence of the PKCα-inhibitor GÖ6976 (20 µM) (c, f) (scale bars 10 µm). (B, C) Nephrin expressed at the surface of murine PKCα+/+ and PKCα−/− and human podocytes is labelled with an anti-nephrin antibody at 4°C. (B) For temperature shift experiments cells were left at 4°C or incubated at 37°C for 20 min in the absence or presence of the PKCα-Inhibitor GÖ6976 (20 µM) to induce internalization. (C) For glucose experiments murine PKCα+/+ and PKCα−/− and human podocytes are stimulated for 1 hour with high glucose (30 mM) or Mannitol. Endocytosis of nephrin is stopped by cooling down at 4°C and labelled with an anti-nephrin antibody. Extinction was measured at 405 nm. Bar graphs represent the graphical summary of three independent experiments. *p<0.035, compared by Student's t-test for temperature shift and glucose experiments. (D) *PKCα overexpression enhances nephrin endocytosis*. HEK 293T cells were transfected with mNephrin-flag or hNephrin-SV5 and PKCα-GFP, or with the empty vector GFP-N1. Nephrin expressed at the surface is labelled with an anti-flag or anti-SV5 antibody. The cells are labelled at 4°C followed by an incubation at 37°C for 20 min in the absence or presence of the PKCα-inhibitor GÖ6976 (20 µM) to induce internalization. The extinction was measured at 405 nm. Bar graphs represent the graphical summary of three independent experiments. ^*^ P<0.002, compared by Student's *t-test*. Mouse or rabbit IgG was used to exclude non specific surface binding of the primary antibody. (E) Cell surface was biotinylated in PKCα+/+ and PKCα−/− podocytes before and after endocytosis induction. Cells were lysed and biotinylated proteins were precipitated using streptavidin-beads. Beads were boiled in loading buffer and western blot procedure revealed nephrin content of biotinylated protein fraction. Lysates were analyzed for nephrin. Bar graphs show the graphical summary of two independent experiments, (*p<0.03) compared by *Student's t-test*.

Since our results so far indicated a direct protective effect of PKCα inhibition on nephrin expression and proteinuria development we wanted to delineate the underlying mechanisms. Nephrin is a key component of the glomerular slit diaphragm. It is a transmembrane protein (185–200 kDa) with a short intracellular domain, and an extracellular domain with eight distal IgG-like motifs. Nephrin molecules of neighbouring foot processes build a zipper-like structure that makes up the last barrier for serum proteins to the urine [10]. In addition, nephrin is a platform of signaling proteins important for the integrity of the podocyte cytoskeleton and for the activation of signaling cascades such as PI3K/AKT and ERK1/2 that control survival pathways of the podocyte [11]. Mutations of the nephrin gene lead to congenital nephrotic syndrome with proteinuria and renal failure [12]. Thus far, only little is known about the normal protein turnover of slit diaphragm proteins. Recently, *Quack et al.* described a β-arrestin mediated mechanism of nephrin endocytosis as a molecular basis for the turnover of slit diaphragm molecules [13]. Moreover, *Quin et al.* demonstrated that nephrin can be internalized via raft-mediated endocytosis [14]. PKCα has been previously described to regulate caveolar endocytosis [15],[16] and to be involved in endocytosis of transmembrane receptors [17], [18]. On the basis of the published evidence and our results it was tempting to speculate that PKCα could be a signaling mediator to orchestrate nephrin endocytosis in podocytes. Therefore, we performed an immunofluorescence-based endocytosis assay in murine PKCα-wildtype (PKCα+/+) and PKCα-knockout (PKCα−/−) podocytes in culture. When we examined endocytosis of surface expressed nephrin we could demonstrate that nephrin is endocytosed in PKCα+/+ podocytes after 20 min temperature shift to 37°C. Nephrin became sorted into larger vesicles and distributed from the cell periphery to the perinuclear compartment. Furthermore, nephrin colocalized with the raft/caveolar-mediated endocytosis marker Cholera toxin B (Fig. 4A, panel b). In contrast to that, we could demonstrate that nephrin endocytosis is almost absent in PKCα+/+ podocytes in the presence of the PKCα inhibitor GÖ6976 and in PKCα−/− podocytes (Fig. 4A, panels c and e). The general uptake of Cholera toxin B is not inhibited by inhibition of PKCα since intracellular accumulation of green vesicles as indicated by Cholera toxin B uptake in the presence of GÖ6976 or in the absence of PKCα is clearly visible (Fig. 4A, panel c and e). This would indicate a partially specific effect for nephrin endocytosis, however a similar effect on other transmembrane proteins cannot be excluded. To exclude non-specific surface binding or uptake of secondary antibody we performed the temperature shift in the absence of primary antibody and we could not detect a significant red fluorescence signal (Supplementary Fig. S2). So in summary, our Cholera toxin B experiments indicate a PKCα mediated effect on nephrin endocytosis, however for a clear differentiation of raft- or clathrin-mediated endocytosis additional experiments would be required since Cholera toxin B cannot clearly differentiate both endocytosis types.

To quantify nephrin endocytosis we used an observer independent assay system with a cell-based ELISA. We could demonstrate a reduced rate of nephrin endocytosis in cultured murine PKCα+/+ and human podocytes in the presence of GÖ6976 and in murine PKCα−/− podocytes after temperature shift to 37°C (Fig. 4B). Furthermore, high glucose treatment for 1 hour leads to endocytosis of nephrin in murine PKCα+/+ and human podocytes. This effect is significantly higher compared to high glucose treated PKCα+/+ and human podocytes in the presence of GÖ6976 and in PKCα−/− podocytes (Fig. 4C). To test whether PKCα overexpression would lead to an increased rate of nephrin endocytosis we performed overexpression experiments in HEK293T cells. In this artificial overexpression system we could also document reduced endocytosis of overexpressed mouse and human nephrin constructs in the presence of GÖ6976. Interestingly, we found that the simultaneous overexpression of a PKCα-construct would lead to an enhanced nephrin endocytosis response. This effect was partially inhibited in the presence of the PKCα inhibitor (Fig. 4D). In addition, we could confirm these results in PKCα+/+ and PKCα−/− podocytes using surface biotinylation before and after nephrin endocytosis. We again detected a higher amount of biotinylated nephrin in PKCα−/− podocytes indicating a higher rate of endocytosis in the wildtype cells (Fig. 4E). To exclude non specific surface binding of the primary antibody we performed the experiments with a mouse or rabbit IgG control (Santa Cruz, CA, USA). To ensure that our results in the different cell types were not influenced by reduced cell survival during the temperature shift or transfection procedures we used trypan blue stainings on all cell types to proof cell viability. None of the temperature conditions would affect cell viability in the murine and human podocyte cell lines or the transfected HEK293T cells (Supplementary Fig. S3).

### PKCα regulates nephrin endocytosis via PICK1

**Figure 5:**
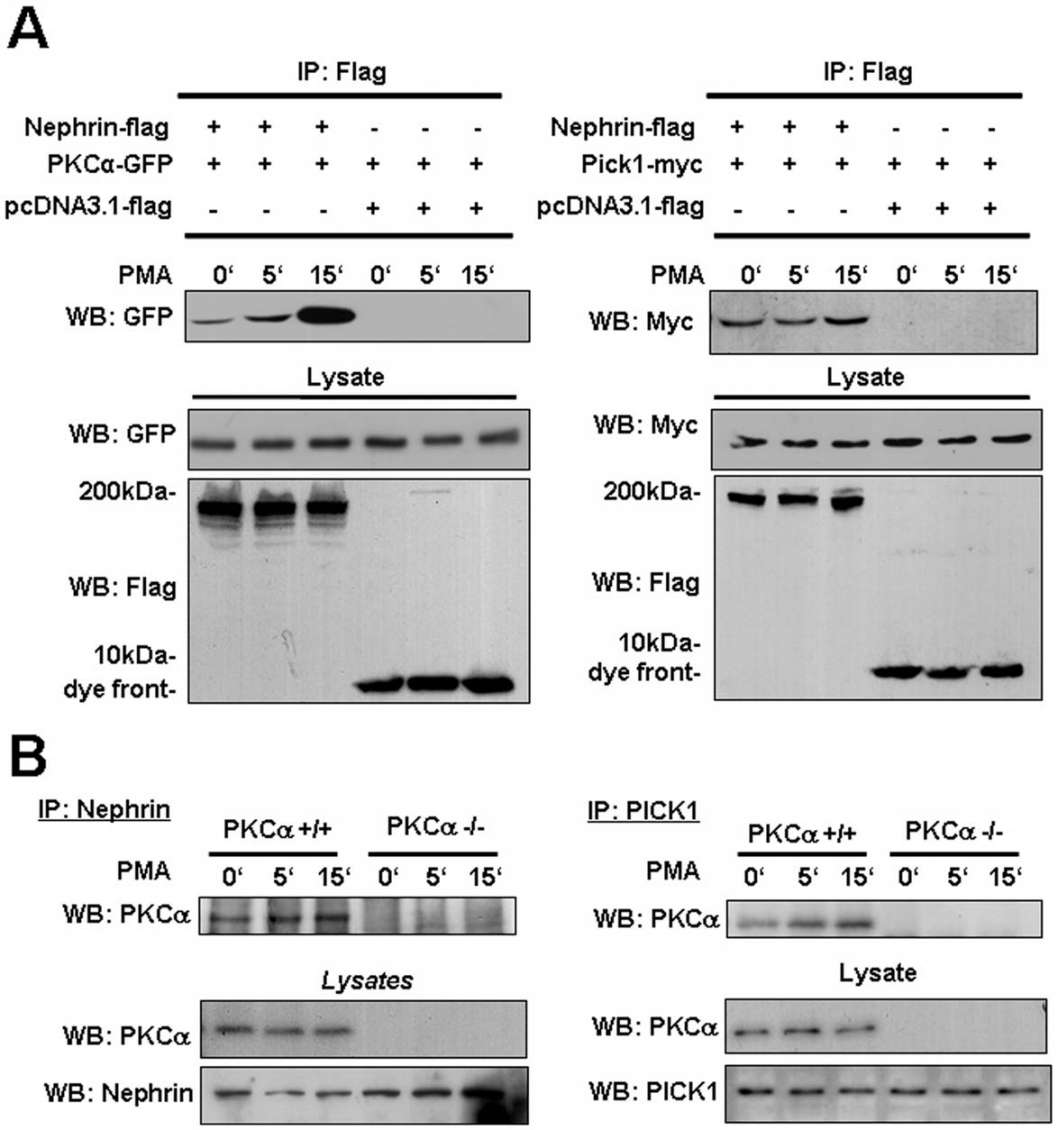
Inducible interaction of transiently overexpressed and endogenous nephrin with PKCα and PICK1. *(A)* HEKT293T cells were transiently transfected with mNephrin-flag, PKCα-GFP and PICK1-myc and stimulated with PMA for 0, 5 and 15 min. Nephrin-flag was precipitated with agarose labelled with an anti-Flag-antibody. The probes were blotted and analyzed for GFP and Myc. (B) Endogenous nephrin and PICK1 were precipitated (IP) using an anti-nephrin and anti-PICK1 antibody from whole cell lysates of PKCα+/+ and PKCα−/− podocytes which were treated for 0, 5 and 15 min with PMA. The probes were blotted and analyzed for PKCα, nephrin and PICK1.

To understand the observation of reduced proteinuria and preserved nephrin expression mechanistically, we investigated whether PKCα interacts with nephrin and whether that interaction would lead to nephrin internalization. Using co-immunoprecipitations we could demonstrate a PMA inducible interaction of PKCα and murine nephrin in transfected HEK293T cells (Fig. 5A). PKCα binds to nephrin and we detected a strong inducible interaction within 15 minutes. A similar result was obtained when we used human nephrin in our experiments (Supplementary Fig. S4). Since PKCα dependent endocytosis has been described earlier in orchestration with the PDZ- and BAR-domain-containing protein PICK1 (protein interacting with C-kinase) [19], [20], we wanted to test whether PMA stimulation would lead to recruitment of PICK1 to this complex. Similarly to our results with PKCα, we could detect a PMA inducible interaction of nephrin and PICK1 (Fig. 5A). To confirm our results on an endogenous level, we performed co-immunoprecipitations of endogenous nephrin and endogenous PKCα in podocytes. Similarly to our results in HEK293T cells, we found an inducible interaction of PKCα and PICK1 within 15 minutes after stimulation with PMA (Fig. 5B) in PKCα+/+ podocytes. In contrast, no association was detectable in the PKCα−/− podocytes indicating an endogenous protein complex specifically orchestrated via PKCα (Fig. 5B). In summary, our data indicate that PKCα expression can be regulated by different stressors in podocytes *in vitro* and *in vivo*. Specifically in the diabetic millieu PKCα mediates nephrin endocytosis and PKCα-deficiency or inhibition leads to preserved nephrin expression on the podocyte surface and thus has a direct protective effect on the integrity of the slit diaphragm.

## Discussion

Our results provide novel mechanistic insights into the relationship of PKCα activity and the signaling response of podocytes under diabetic conditions. Nephrin is a single transmembrane receptor that belongs to a family of adhesion molecules. A genetic defect in nephrin expression leads to congenital nephrotic syndrome [21]; [22]. Furthermore, nephrin signaling contributes to the overall survival response in podocytes and anchors important signaling molecules to the glomerular filtration slit [11]. Nephrin molecules of neighbouring foot processes built the backbone of the slit diaphragm and together with the recently identified Neph1 it forms a hetero-oligomeric complex in the plane of the membrane that interacts across the foot process interaction [23], [24]. Since the blood flow dependent changes of the glomerular capillary diameter require constant adaptation processes, it seems likely that foot process formation and the formation of the slit diaphragm is a highly dynamic process with constant turnover and renewal of these transmembrane proteins. A molecular regulation of the slit diaphragm complex turnover via nephrin phosphorylation and endocytosis was first described by *Quack and coworkers* [13]. In this manuscript we describe PKCα as a high glucose inducible kinase that orchestrates nephrin internalization in podocytes. We found a significant induction of glomerular PKCα expression in diabetic glomeruli in podocytes in mice and humans. This stress induced glomerular induction effect in diabetes is novel. A regulation of PKCα/β in podocytes has previously only been documented in human membranous nephropathy, however the conclusion of its functional role remained elusive [25]. Strikingly, when we injected mice with a PKCα inhibitor we observed a preserved nephrin expression which is reflected in the urine by a significantly reduced excretion of albumin. This is in line with previous observation from our group where we detected preserved nephrin expression and reduced proteinuria in diabetic PKCα deficient mice [5]. In addition, we can demonstrate a glucose dependent induction of PKCα mRNA and protein in murine and human podocytes. Moreover, we demonstrate in different *in vitro* experiments PKCα-mediated endocytosis of murine and human nephrin. *Qin and coworkers* showed a raft-mediated endocytosis of nephrin [14] This is in line with our observations where we can demonstrate that PKCα-mediated endocytosis of nephrin is raft-mediated. Many other studies showed that raft-mediated endocytosis by caveolar are PKCα-regulated [15],[16]. Supporting evidence comes from our immunoprecipitation findings, where we demonstrate that PKCα associates with nephrin and PICK1 after PMA stimulation. Recruitment of PICK1 has been demonstrated as a key step in PKCα mediated trafficking of AMPA receptors via direct interaction with PKCα and the AMPA receptor subunits GluR1-GluR4 [19] in the PDZ domain of PICK1. This association is required for AMPA-receptor trafficking. This inducible interaction of endogenous nephrin and endogenous PKCα is reproducible in transient assays as well as with endogenous proteins in podocytes. On the background of these results we speculate that PKCα activity contributes to the pathogenesis of proteinuria by disrupting the glomerular filter in response to high glucose via nephrin internalization. Absence or inhibition of PKCα leads to stabilized surface expression of nephrin and this has positive effects on proteinuria development. We would further hypothesize that nephrin or possibly turnover of the whole slit diaphragm complex is regulated similar as turnover and trafficking of activated growth factor receptors [13]. This would support the role for nephrin as a structural sensor regulating the integrity of the slit diaphragm.

In diabetes microalbuminuria is one of the earliest detectable abnormalities that, when left untreated, will eventually progress to gross proteinuria. Since the degree of proteinuria correlates with tubular interstitial fibrosis the early treatment of proteinuria and prevention of progression to significant proteinuria levels is an important step to delay disease progression. In the development of diabetic glomerulopathy an increase in podocyte foot process width and podocyte loss has been observed in a variety of patients with diabetes [26], [27]. Also, loss and downregulation of nephrin is a well described effect in patients with diabetic nephropathy compared to healthy controls [28].

In summary, our results open new avenues for further investigations on slit diaphragm turnover under physiological and pathological conditions. Furthermore, PKCα inhibition is a promising target molecule for podocyte protective therapy in proteinuric kidney diseases and in particular for diabetic nephropathy.

## Supporting Information

Figure S1 Podocytic nephrin mRNA expression is not altered by high glucose treatment. Q-PCR for Nephrin in murine and human podocytes demonstrates mRNA expression in a time course experiment after stimulation with high glucose (30 mM) and mannitol as osmotic control for up to 24 hrs (results are representative for 3 independent experiments).
(0.92 MB TIF)

Figure S2 Staining procedure with rabbit IgG. PKCα+/+ podocytes were double labelled with Cholera toxin-B (green) and rabbit IgG followed by an Cy3-labelled secondary antibody (red) as a negative control for unspecific binding of the primary and secondary antibody. The cells are labelled at 4°C.
(1.07 MB TIF)

Figure S3 Podocytes and transfected HEK 293T cells are viable during endocytosis assay. PKCα+/+, PKCα−/−, human podocytes and transfected HEK293T cells (hNephrin-SV5 and PKCα-GFP) were cooled down at 4°C, shifted to 37°C in the absence or presence of the PKCα inhibitor GÖ6976 or permeabilized with 80% methanol as control for 10 min. Cells were stained with 0,4% trypan blue.
(2.89 MB TIF)

Figure S4 Inducible Interaction of transiently overexpressed human nephrin with human PKCα. HEK293T cells were transiently transfected with hNephrin-flag and hPKCα-GFP and stimulated with PMA for 0, 5 and 15 min. Nephrin-flag was precipitated with agarose labelled with an anti-Flag-antibody. The probes were blotted and analyzed for GFP and Flag content.
(0.94 MB TIF)
