# Supplementary figures and images for: Podocytic PKC-Alpha Is Regulated in Murine and Human Diabetes and Mediates Nephrin Endocytosis
Source: PLoS One. 2010 Apr 16;5(4):e10185. doi: 10.1371/journal.pone.0010185 (PMC2855708; doi:10.1371/journal.pone.0010185)

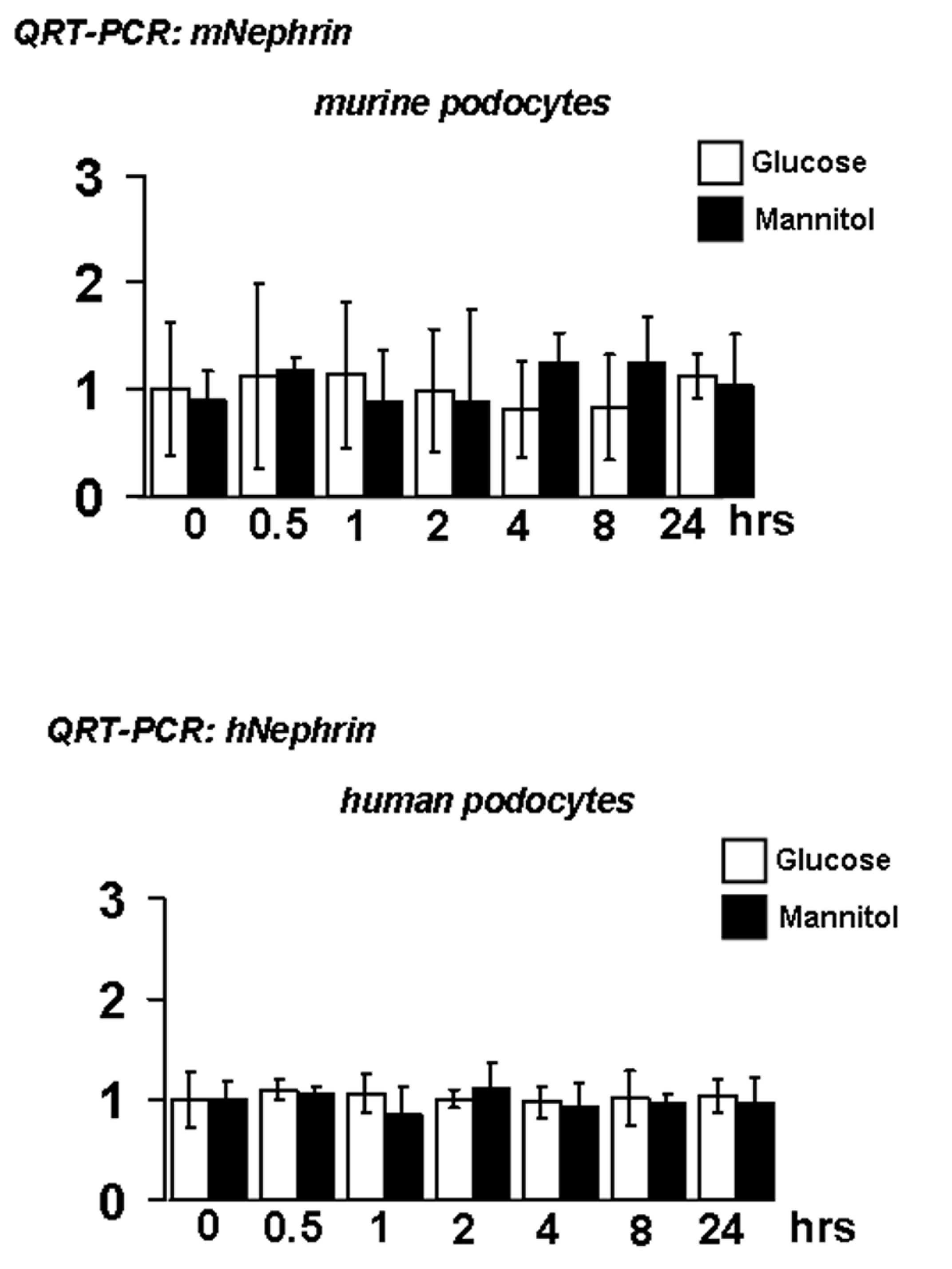

Supplement: Figure S1 — Podocytic nephrin mRNA expression is not altered by high glucose treatment. Q-PCR for Nephrin in murine and human podocytes demonstrates mRNA expression in a time course experiment after stimulation with high glucose (30 mM) and mannitol as osmotic control for up to 24 hrs (results are representative for 3 independent experiments). (0.92 MB TIF) [file pone.0010185.s001.tif]

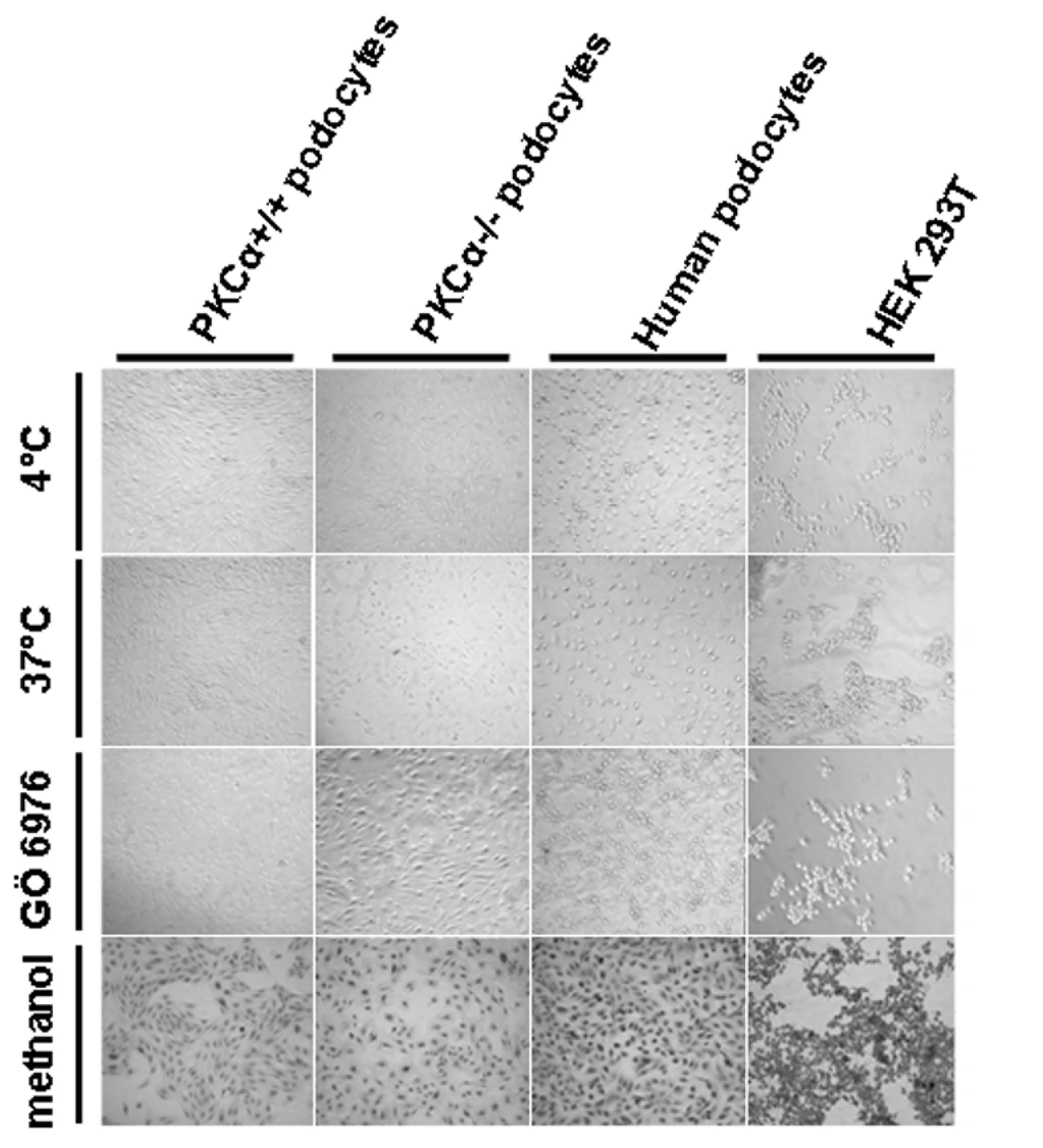

Supplement: Figure S3 — Podocytes and transfected HEK 293T cells are viable during endocytosis assay. PKCα+/+, PKCα−/−, human podocytes and transfected HEK293T cells (hNephrin-SV5 and PKCα-GFP) were cooled down at 4°C, shifted to 37°C in the absence or presence of the PKCα inhibitor GÖ6976 or permeabilized with 80% methanol as control for 10 min. Cells were stained with 0,4% trypan blue. (2.89 MB TIF) [file pone.0010185.s003.tif]

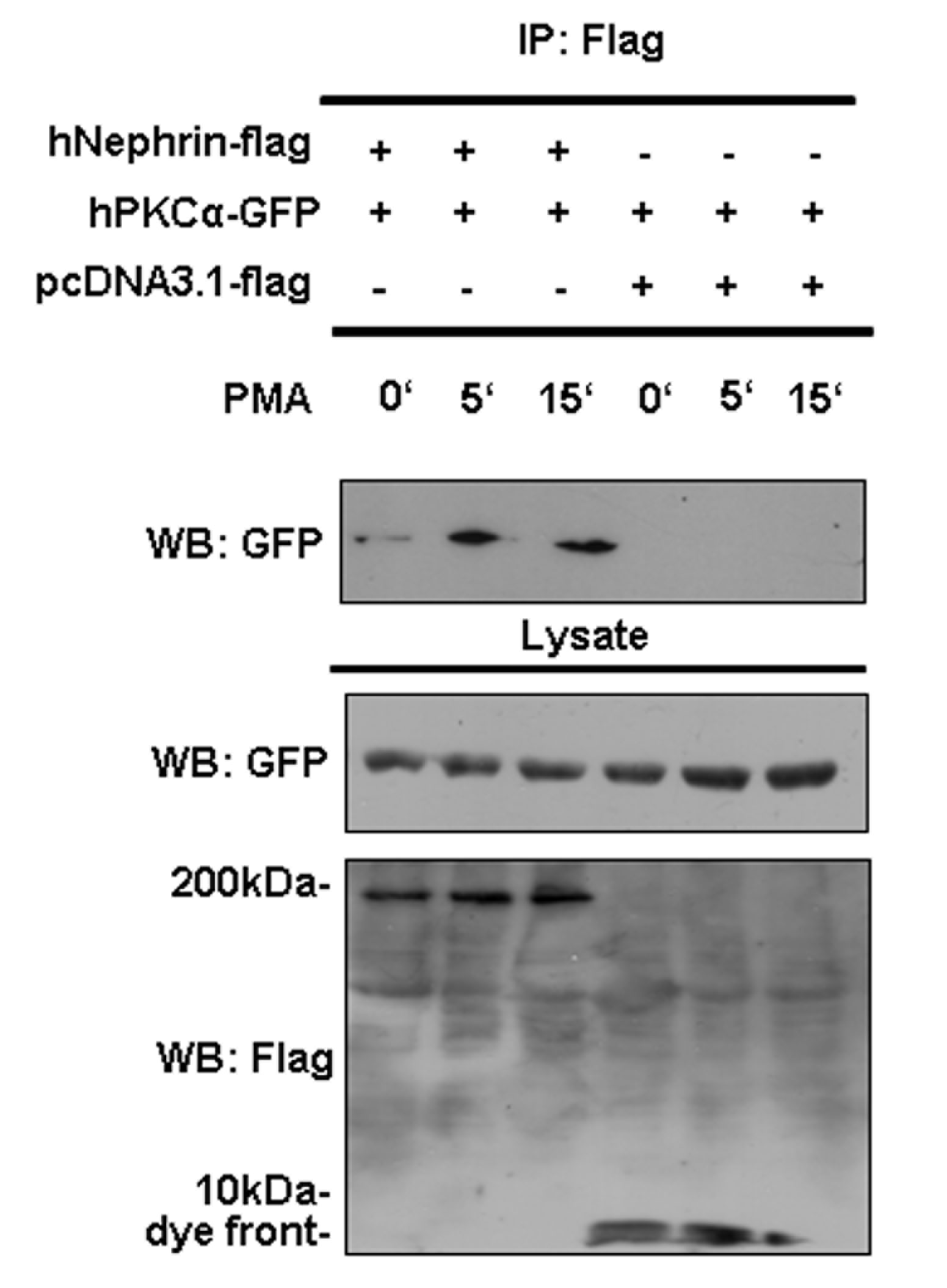

Supplement: Figure S4 — Inducible Interaction of transiently overexpressed human nephrin with human PKCα. HEK293T cells were transiently transfected with hNephrin-flag and hPKCα-GFP and stimulated with PMA for 0, 5 and 15 min. Nephrin-flag was precipitated with agarose labelled with an anti-Flag-antibody. The probes were blotted and analyzed for GFP and Flag content. (0.94 MB TIF) [file pone.0010185.s004.tif]
